# Unraveling the Underlying Molecular Mechanism of ‘Silent Hypoxia’ in COVID-19 Patients Suggests a Central Role for Angiotensin II Modulation of the AT1R-Hypoxia-Inducible Factor Signaling Pathway

**DOI:** 10.3390/jcm12062445

**Published:** 2023-03-22

**Authors:** Christian Albert Devaux, Jean-Christophe Lagier

**Affiliations:** 1 Institut de Recherche pour le Développement, Assistance Publique Hôpitaux de Marseille, Microbes Evolution Phylogeny and Infection Laboratory, Aix-Marseille University, 13000 Marseille, France; 2 Institut Hospitalo-Universitaire—Méditerranée Infection, 13000 Marseille, France; 3 Centre National de la Recherche Scientifique, 13000 Marseille, France

**Keywords:** COVID-19, silent hypoxia, angiotensin II, ACE2, renin-angiotensin system, AT1R, hypoxia-inducible factors, HIF-1, hypoxic spillover

## Abstract

A few days after being infected with SARS-CoV-2, a fraction of people remain asymptomatic but suffer from a decrease in arterial oxygen saturation in the absence of apparent dyspnea. In light of our clinical investigation on the modulation of molecules belonging to the renin angiotensin system (RAS) in COVID-19 patients, we propose a model that explains ‘silent hypoxia’. The RAS imbalance caused by SARS-CoV-2 results in an accumulation of angiotensin 2 (Ang II), which activates the angiotensin 2 type 1 receptor (AT1R) and triggers a harmful cascade of intracellular signals leading to the nuclear translocation of the hypoxia-inducible factor (HIF)-1α. HIF-1α transactivates many genes including the angiotensin-converting enzyme 1 (ACE1), while at the same time, ACE2 is downregulated. A growing number of cells is maintained in a hypoxic condition that is self-sustained by the presence of the virus and the ACE1/ACE2 ratio imbalance. This is associated with a progressive worsening of the patient’s biological parameters including decreased oxygen saturation, without further clinical manifestations. When too many cells activate the Ang II-AT1R-HIF-1α axis, there is a ‘hypoxic spillover’, which marks the tipping point between ‘silent’ and symptomatic hypoxia in the patient. Immediate ventilation is required to prevent the ‘hypoxic spillover’.

## 1. Introduction

Severe acute respiratory syndrome cases were observed in Wuhan (China) in December 2019. This new disease (later named coronavirus disease 2019 or COVID-19) was characterized by acute respiratory distress (ARDS), cytokine storm, and thrombotic events leading to multiple organ dysfunction syndrome (MODS) and a risk of death. Shortly after the recognition of COVID-19, a new Sarbecovirus, the severe acute respiratory syndrome-related coronavirus 2 or SARS-CoV-2, was identified as the etiological agent of the disease [1,2]. Of the 670 million people who have been infected with SARS-CoV-2 since the beginning of the pandemic, a small fraction of those patients have experienced a severe form of the disease, and COVID-19 has been responsible for more than 6.8 million deaths (https://coronavirus.jhu.edu/map.html; accessed on 20 February 2023). Very quickly, angiotensin I-converting enzyme 2 (ACE2), a key modulator of the renin–angiotensin system (RAS)—also known as the renin–angiotensin–aldosterone system (RAAS)—was demonstrated to be the viral entry receptor for SARS-CoV-2 [3,4]. SARS-CoV-2 gains entry into alveolar type II epithelial lung cells [5,6], as well as many other cell types expressing a transmembrane ACE2, by way of its envelope spike (S) glycoprotein, which comprises S1 and S2 subunits. After attachment to ACE2 and S glycoprotein cleavage by serine protease (e.g., TMPRSS2) favoring viral ingress, ACE2 is downregulated though several mechanisms, including the shedding of a soluble form of the ectodomain of the molecule (sACE2) [7].

Upon admission to hospital for a SARS-CoV-2 diagnostic, some SARS-CoV-2-positive patients who described themselves as comfortable (patients do not notice any vital signs of collapsed alveoli and air sacs), required special attention: their clinical examination revealed a clinically silent but physiologically proven decrease in the arterial oxygen saturation of hemoglobin (SaO_2_), and further examination by low-dose computed tomography (LDCT) showed images compatible with pneumonia. This condition is named ‘silent hypoxia’, ‘asymptomatic hypoxia’, or ‘happy hypoxia’. Following ‘silent hypoxia’, untreated COVID-19 patients often develop shortness of breath and dyspnea, which triggers local hypoxia. The prevalence of ‘silent hypoxia’ in COVID-19 patients ranges from 20% to 40% [8,9,10]. This ‘silent hypoxia’ is associated with a very poor outcome, since about one third of these asymptomatic patients should then be transferred to the intensive care unit (ICU); moreover, about one quarter of patients who were transferred to the ICU died from the disease [11]. This simple observation makes it necessary to understand the underlying mechanism of ‘silent hypoxia’ in an attempt to possibly highlight new targets for the prevention of this asymptomatic pathology. Intervening early enough in this process would avoid a rapid spread of uncontrollable micro-events (e.g., microthrombi in the pulmonary vasculature) promoting multifocal microthrombotic interstitial pneumonitis associated with a slight/moderate intra-alveolar exudate preceding multi-organ dysfunction (including lungs, heart, kidneys, and intestines) [12,13,14].

If the development of COVID-19 has brought forth the phenomenon of silent hypoxia, this decrease in SaO_2_ is a common finding in medicine and is usually precipitated by respiratory disorders or cardiac diseases. It can be observed with diseases such as chronic obstructive pulmonary disease (COPD), pulmonary embolus, atelectasis (collapse of the lung), intrapulmonary shunting (e.g., pulmonary arterivenous malformation, hepatopulmonary syndrome), intracardiac shunting (e.g., atrial or ventricular septal defects), neurological injury-associated hypoventilation, and obesity-hypoventilation syndrome. Alveolar hypoxia causes hypoxic pulmonary vasoconstriction (HPV) by means of mechanisms local to the lung [15]. The most common mechanism of hypoxia is perfusion through areas that are not well ventilated as a consequence of ventilation–perfusion mismatch (imbalance between the volume of gas expired by the alveoli and the pulmonary capillary blood flow) [16]. Another important cause of hypoxia is left-to-right shunt—a condition in which there is a transfer of blood from the left side of the heart to the right side as a result of a hole in the walls separating the upper chambers (atrium) or lower chambers (ventricles) [17,18]. Hypoxia can also be caused by right ventricular dysfunction following pericardiocentesis [19]. Intraoperative hypoxia following atelectasis is sometimes associated with surgery for abdominal pathology or hip arthroplasty, laryngospasm, and/or inhalational anesthetics [20].

## 2. Clinical Evidence of ‘Silent Hypoxia’

Obviously, the ability to maintain O_2_ homeostasis is essential for human survival. Up until now, ‘silent hypoxia’ has been one of the most life-threatening medical problems of COVID-19 [10]. Despite a lack of dyspnea on admission, those patients have hypoxemia/hypocapnia syndrome and are at a high risk of seeing their state of health deteriorate rapidly with irreversible damage to vital organs if left undetected for too long without clinical management (using ventilation with NO gas to improve their SaO_2_) [10,11,21]. ‘Silent hypoxia’ was usually associated with advanced age and comorbidities [22]. Phenotypic differences were observed among patients according to lung elastance, lung recruitability, ventilation-to-perfusion ratio, right-to-left shunt, as well as stress, pain, fear, and anxiety.

The early detection of silent hypoxia in COVID-19 patients is crucial to minimize the risks for severe forms of COVID-19, such as COVID-19-related acute respiratory distress syndrome (C-ARDS), as well as to reduce mortality rate [11,23]. Several reports have revealed that of the COVID-19 patients presenting at the Emergency Department with a SpO_2_ < 90%, the prevalence of silent hypoxia in these patients ranges from 20% to 40%, and that patients with age >65 years, male sex, high body mass index, cardiac disease, COPD, and high D-dimer are more likely to present with silent hypoxia [10,22,24]. In the Wuhan cohort, 62.4% of severe COVID-19 cases and 46.3% of those who were eventually intubated and ventilated exhibiting sinus tachypnea (increase in normal breathing rate) and higher inflammatory marker levels, or who died, did not present dyspnea [25,26,27]. Similar observations of shortness of breath have also been reported by other clinicians who suggested the possibility of SARS-CoV-2-induced ACE2 positive neuronal cells death in areas devoted to the sensory perception of dyspnea, or that the cytokine storm associated with the Ang II-AT1R pathway leads to indirect toxic effects on the corticolimbic network [28,29].

In the absence of a prompt medical decision, there is a progressive increase in the occurrence of microvascular thrombi preventing gas exchange in the normally oxygenated areas of tissues leading to the sudden development of hypoxemia in COVID-19 patients. In addition to the formation of fibrin thrombi in tissues where inflammatory cells and platelets are present in abundance, there is also an increased alveolar capillary permeability and exudation into the alveoli, as well as coagulation factors, including fibrinogen. Elevated D-dimers (a biomarker of fibrin degradation) upon patient admission indicate the existence of hypercoagulation and pulmonary embolisms and are associated with increased mortality in severe COVID-19 patients [30,31,32,33]. Venous thromboembolism is frequently found to be associated with SARS-CoV-2 infection [34,35,36]. Therefore, in the most severe forms of the disease, patients suffer from acute respiratory distress syndrome (ARDS) with dyspnea and abnormally ventilated areas of the lungs, resulting in hypoxemia, which has been proven to be associated with hospital mortality [37,38,39]. This easily recognizable symptomatic phase is likely preceded by an asymptomatic phase (‘silent hypoxia’), during which the molecular crosstalk between the molecules of the RAS orchestrated by the circulation of the virus, free S1 and S1/sACE2 complexes predisposes to the establishment of the symptomatic phase. It has been suggested that ‘silent hypoxia’ is caused by a combination of biological dysfunctions affecting the lungs of COVID-19 patients. These pathological processes include microvascular thrombosis, pulmonary embolisms, ventilation–perfusion mismatching in the non-injured lung, capillary flow redistribution, air sac collapse (atelectasis), flow through intrapulmonary arteriovenous anastomoses, and normal perfusion of the relatively small fraction of injured lungs [40,41,42]. Before reaching this stage, the pathological process is set up insidiously by deregulation signaling at the level of certain cells (e.g., type II pneumocytes), and then amplified in the microenvironment of those cells. In the initial phase of infection, the virus can enter the peripheral blood stream via the lungs and spread to other organs.

Interestingly, chest computed tomography (CT)-scan abnormalities in people infected with SARS-CoV-2 but who are asymptomatic or pauci-symptomatic have been largely observed, thanks to the wide use of low-dose CT-scans [11]. Most of the patients had some CT-scan abnormalities, mainly as exclusive ground-glass opacities or erratic paving patterns. A key to the good management of COVID-19, especially in patients with risk factors, was to obtain regular oxygen saturation readings by pulse oximetry to enable the early diagnosis of ‘silent hypoxia’ and, consequently, to achieve more appropriate management, including early oxygen supplementation to reduce poor clinical outcomes [43,44]. Although in our experience it has been beneficial for patients, the benefit of adding home pulse oximetry for the remote monitoring of COVID-19 patients is still debated [45]. Regardless, it is imperative that patients be placed under strict day-to-day monitoring [11]. In addition to a pulse oximeter, the other diagnostic tools suitable for identifying the presence of silent hypoxia consist of measuring the arterial blood gas levels and D-dimer [42]. The early detection of silent hypoxia may facilitate the decision on when to start the oxygen treatment or whether to involve an invasive or non-invasive method of oxygenation.

## 3. Regulation of Blood Pressure Homeostasis via the RAS

The angiotensin-converting enzyme 2 (ACE2), which is expressed in a large number of tissues, including endothelial cells of the arteries, arterioles, and venules of the heart and kidney, plays a crucial role in maintaining blood pressure homeostasis, and thus, regulates the pulmonary vascular tone. An imbalance in blood pressure might lead to the development of vascular remodeling and vascular rigidity, which predisposes patients to heart left-ventricular hypertrophy and fibrosis, acute kidney injury, and extensive microthrombosis in coronary and pulmonary circulation [46].

**Figure 1:**
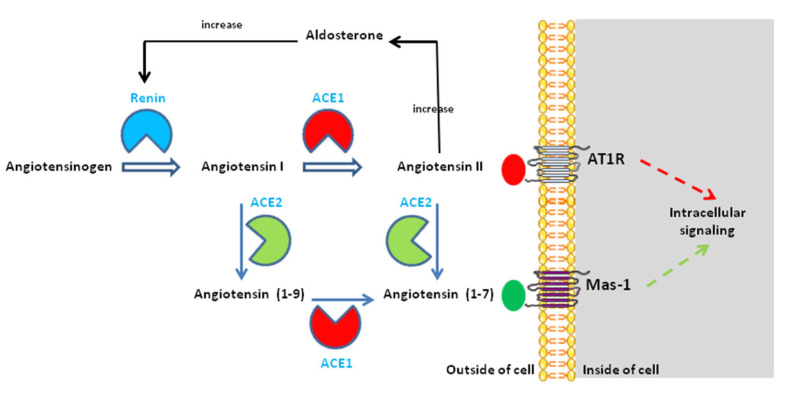
Structure of the renin–angiotensin–(aldosterone) system and the role of ACE2 in this physiological system. ACE1 catalyzes the conversion of angiotensin I (Ang I) to angiotensin II (Ang II). ACE2 catalyzes the conversion of Ang I to angiotensin-(1-9) and the conversion of Ang II to angiotensin-(1-7). There are also known interactions between the RAS and the Kininogen–(kallikrein)–Bradykinin system (not shown). Under normal physiological conditions, ACE2 converts Ang II into Ang-(1-7), which exhibits vasodilatory, anti-proliferative, and anti-inflammatory effects via the G protein-coupled receptor called Mas-1. Ang-(1-7) counterbalances the vasoconstrictor and inflammatory effect of Ang II. Upon SARS-CoV-2 entry, the downregulation of ACE2 decreases its ability to generate angiotensin (1-9) from Ang I and angiotensin-(1-7) from Ang II, leading to renin–angiotensin system (RAS) imbalance and overactivation of the Ang II -AT1R axis. For more details, see reference [70].

It has been known for decades that ACE2 regulates the balance of two major mediators: angiotensin II (Ang II) and angiotensin-(1-7) (Ang-(1-7) (Figure 1). The regulation of blood pressure by ACE2 is obtained by counteracting the action of ACE1, which hydrolyzes the angiotensin 1 to form Ang II (a mediator of vasoconstriction), while ACE2 transforms the neosynthesized pool of Ang II into Ang-(1-7) (a vasodilator and anti-fibrotic molecule). Ang II is known for enhancing inflammation, fibrosis, and endothelial damage. Ang II binds to the Ang II type I and type II receptors (AT1R and AT2R) [47,48,49,50], which are receptors with opposite effects. AT2R is poorly expressed compared to AT1R; therefore, AT1R serves as the major receptor for Ang II-induced cell signals [51,52]. The activation of the Gq coupled receptor AT1R by Ang II is transient and, after phosphorylation by kinases such as PKC and GRKs [53,54,55,56], AT1R internalizes through a mechanism that involves β-arrestin 2, the adaptor protein complex 2 (APC2), clathrin, and intersectin 2 [51,57,58]. Through binding to AT1R, Ang II is a potent activator of NADPH oxidase (NOX) and an inducer of reactive oxygen species (ROS) triggering oxidative injury [59]; it also induces proinflammation [60,61,62,63]. While many NOX enzymes are localized at least in part at the cell surface where they can release ROS into the extracellular milieu, NOX4 is mainly localized to the endoplasmic reticulum and mitochondria [64]. NOX4, which functions as a constitutively active O_2_ sensor, generates H_2_O_2_ as a function of the cellular oxygen concentration (pO_2_). The production of ROS diverts the cytoplasmic pool of HIF-1α from being degraded through the proteasome under control of the prolyl hydroxylase (PHD) and the von Hippel-Lindau gene product (pVHL) E3 ubiquitin ligase, two molecules in charge of maintaining homeostasis [65]. Under Ang II signal, HIF-1α is translocated to the cell nucleus where it modulates the expression of several genes. Furthermore, Ang II inhibits the production of nitric oxide (NO), leading to vascular injury [66]. Finally, the interaction of Ang II with AT1R induces a cascade of events (Rac1, NOX4, ROS, MEK, ERK1/2) leading to the phosphorylation of the p65 subunit of NF-κB, which in turn activates the production of pro-inflammatory molecules [67,68,69]. 

Within the molecular cascade devoted to maintaining blood pressure homeostasis, ACE2 is not the unique effector able lead to the production of Ang-(1-7). Several peptidases, including neprilysin (NEP), vascular endothelium prolyl peptidases, and smooth muscle thimet oligopeptidase, can catalyze the production of Ang-(1-7) [71]. Ang-(1-7) binds to the G protein-coupled receptor (GPGR) named Mas 1 and is characterized by its vasodilating properties and its anti-proliferative and anti-inflammatory effects [72,73,74]. The activation of MAS 1 induces nitric oxide synthesis.

## 4. The RAS Imbalance in COVID-19

Notably, COVID-19 has a strong vascular component, with microthrombosis events and exacerbated effects on the microvasculature comprising the arterioles, capillaries, and venules. The RAS imbalance determines the clinical outcome of COVID-19 [60,75]. The fact that SARS-CoV-2 enters susceptible human cells by interacting with ACE2 and TMPRSS2 on the surface of lung type II alveolar cells is the central element of the process that leads to RAS imbalance. The down-modulation of ACE2 following its interaction with Sarbecoviruses has been largely documented in vitro [76,77]. The control of ACE2 gene expression is a multifactorial and complex process that has been recently reviewed elsewhere [70]. The binding of SARS-CoV-2 spike S1 to ACE2 triggers an ACE2 ectodomain cleavage by the ADAM17 sheddase [78,79,80]. The ACE2 cleavages induce the shedding of cellular ACE2 as a soluble form (sACE2) and the systemic release of the S1/sACE2 complex [81]. Moreover, the binding of SARS-CoV-2 to ACE2 is likely to create a steric hindrance at the catalytic site of ACE2, which prevents it from functioning as a peptidase. The inactivation of ACE2 peptidase should lead to the accumulation of Ang II. This is exactly what we previously reported after studying the expression of ACE2 and Ang II in different groups of patients infected with SARS-CoV-2 [82]. We found that COVID-19 patients expressed less ACE2 mRNA, had a lower concentration of plasma sACE2, and had increased plasma levels of Ang II [82]. Although there is still a debate regarding this finding, it was corroborated by most of the other studies that found high increased plasma levels of Ang II in patients with severe COVID-19 [83,84,85]. This suggests that by decreasing the expression of ACE2 and increasing the number of S1/sACE2 complexes, it is no longer possible to achieve efficient processing of Ang II into Ang-(1-7). A surprising observation was that despite the accumulation/increase in Ang II and the reduction in the capacities of the transformation of Ang II into Ang-(1-7) by ACE2, we found that the plasma level of Ang-(1-7) remained almost constant [82]. Another clinical case report described the same observation with severe COVID-19 patients [86]. The plasma level of Ang-(1-7) can be explained either by the availability of a greater quantity of Ang II to undergoing hydrolysis and producing Ang-(1-7), and/or the action of vascular endothelium prolyl peptidases, neprilysin and smooth muscle thimet oligopeptidase to produce Ang-(1-7) directly from Ang I.

## 5. The Ang II-AT1R-HIF-1α Axis

**Figure 2:**
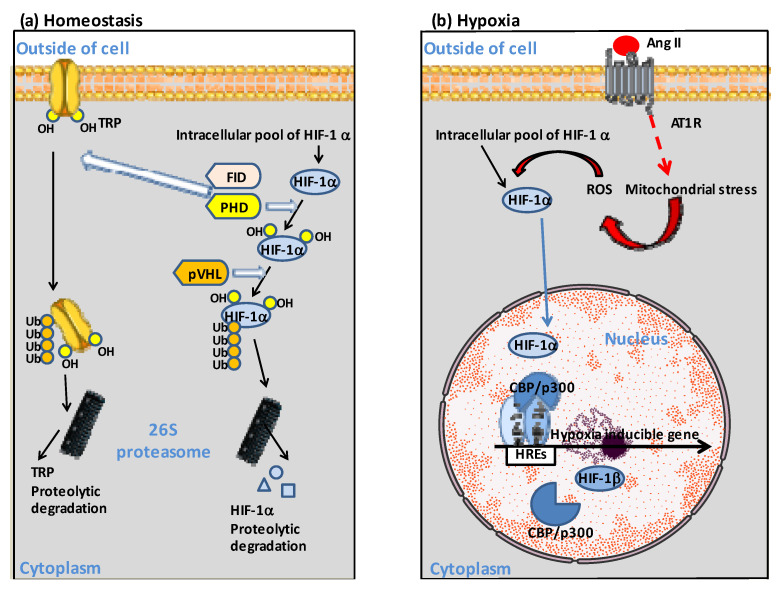
Schematic diagram illustrating the dual regulation of HIF-1α. (**a**) At normal O_2_ concentration, HIF-1α is hydroxylated by the prolyl hydroxylase (PHD). The ubiquitin ligase VHL targets HIF-1α-OH for polyubiquitinylation and proteosomal degradation. Similarly, hydroxylation of the transient receptor potential channel (TRP) by PHD, and asparaginyl hydroxylase FIH targeting TRP-OH for ubiquitinylation and proteosomal degradation, contribute to homeostasis. (**b**) Ang II can contribute to hypoxia through binding to AT1R, which initiates signaling events including activation of reactive oxygen species (ROS) by mitochondria. Under hypoxia, HIF-1α translocates to the cell nucleus where it forms heterodimers with the HIF-β subunit and binds to the hypoxia response element (HRE) in the promoter of hypoxia-inducible genes, recruiting histone acetyltransferases CREB Binding Protein (CBP)/p300 to modulate hypoxia-inducible genes expression. For more details, see reference [65].

Under conditions of RAS imbalance and abnormally high plasma concentrations of Ang II, Ang II binds to AT1R and induces an increased production of ROS via the NADPH oxidase, diminishing nitric oxide (NO) bioavailability. [87,88,89]. Thus, Ang II behaves as a mediator of oxidative stress, in which reactive oxygen species (ROS) are important signaling intermediates in several signal transduction pathways [90]. This oxidative stress prevented HIF-1α from entering its pathway of degradation through the proteasome. HIF-1α translocates to the cell nucleus and forms a heterodimer with the constitutively expressed HIF-1α to activate gene transcription [91,92]. HIF binds to hypoxia-regulated elements (HREs) in the promoter region of hypoxia-inducible genes as an α/β heterodimer forming a complex with the histone acetyltransferases CBP/p300 [93,94,95] (Figure 2).

Several genes are modulated by HIF, including ACE1, myoglobin, endothelial NO synthase, heme oxygenase 2, prolyl hydroxylase 2, FAM213A, lung surfactant protein D, and HIF genes [65,96,97,98]. HIF-1α also activates the transient receptor potential channel (TRP) ankyrin repeat (TRPA1), which leads to intracellular calcium increase, cytokine release modulation, and cell injury [99,100,101,102]. In addition, AT1R signaling modulates Ang II deleterious effects through the activation of multiple downstream pathways (e.g., ERK1/2, NF-κB), some of which lead to the production of specific proinflammatory cytokines (IL-6, IL-1, TNF-α) activation, causing tissue damage [103]. Among these downstream mediators, AT1R is capable of inducing NF-κB, disintegrin, and metalloprotease 17 (ADAM17), altogether promoting the production of TNF-α [104]. This excessive and harmful production of cytokines is known as cytokine storm [105,106]. For example, Liu and colleagues reported a 67.9% increase in IL-6 in patients with COVID-19 and found that levels of IL-6 and C-reactive protein (CRP, a biomarker of inflammation) in the severe group was significantly higher than in the mild group [107].

**Figure 3:**
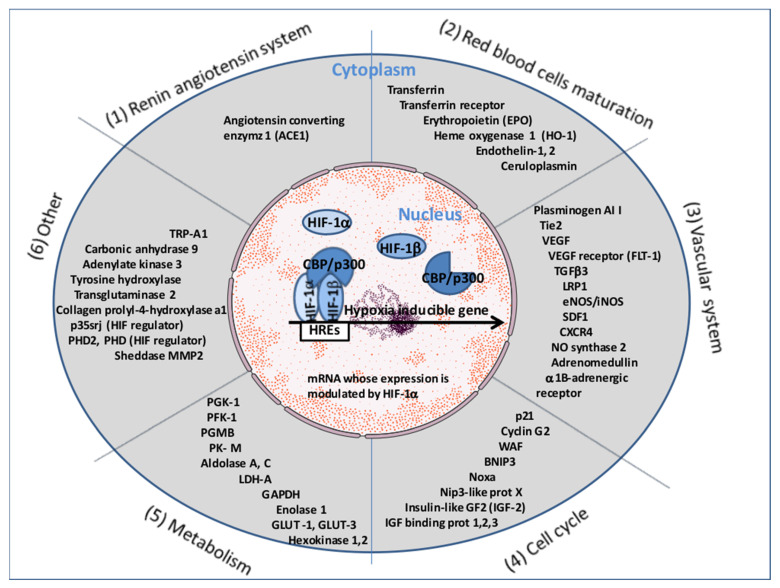
Representative list of cellular genes upregulated by HIF-1. HIF-1α is considered a ‘master regulator’ type of transcription factor capable of controlling the expression of a multitude of genes grouped under the heading of hypoxia-inducible genes α (the list is not exhaustive and grows continuously). The consensus DNA sequence for HIF-1α/HIF-1β binding is common for many genes upregulated during hypoxia. It is worth noting that several genes involved in the RAS, in the development and functioning of the vascular system (which modulate vascular tone or promote angiogenesis), and in erythropoiesis, belong to this list.

Of course, depending on the cell type with which Ang II comes into contact, the Ang II-AT1R-HIF-1α signaling cascade may lead to the expression of a specific subgroup of genes. The consensus DNA sequence for HIF-1α/HIF-1β binding is common for many genes upregulated during hypoxia. It is worth noting that several genes involved in the renin–angiotensin system, in the development and functioning of the vascular system (which modulate vascular tone or promote angiogenesis) and in erythropoiesis, belong to this list (Figure 3). HIF-1α is also known to upregulate p35srj. The p35srj protein is an alternatively spliced isoform of MRG1 which inhibits HIF-1 transactivation by blocking the HIF-1α/p300 interaction. HIF-1α triggers expression of the tyrosine kinase with immunoglobulin and the epidermal growth factor homology domain (Tie2) gene in human coronary microvascular endothelial cells (HCMECs) [108]. The expression of Tie2 (the receptor for angiopoietin 1 and 2) was found to be elevated in COVID-19 patients and may contribute to the angiogenic response in ischemic tissues [109]. Moreover, the Ang II-AT1R interaction also elicits the ADAM17metalloprotease-dependent transactivation of the epidermal growth factor (EGFR) by the heparin-binding EGF-like growth factor (HB-EGF), leading to tyrosine phosphorylation of multiple proteins and the activation of mitogen-activated protein kinase (MAPK/ERK) in various cell types, including vascular smooth muscle cells (VSMC) and hypertrophic responses with a direct action on vascular remodeling [110] (Figure 3).

Moreover, both ACE1 and Ang II play roles in the HIF-1α activation associated with pulmonary hypertension and vascular remodeling [111,112,113]. The Ang II/AT1R pathway was found to induce marked ACE1 upregulation and ACE2 downregulation in patients with hypertensive cardiopathy associated with the activation of the ERK1/2 and p38 MAP kinase [114,115,116,117]. It has been known for many years that HIF-1α is phosphorylated by p42 and p44 mitogen-activated protein kinases (MAPKs also known as ERK1/2), as opposed to by p38 MAPK inducing an upper shift migration of this protein at 12 kDa. It has also been known for a long time that ERK enhances the transcriptional activity of the HIF1 gene [118]. This reinforces the hypothesis that in COVID-19, an excess of Ang II induces signaling via AT1R, which reduces the expression of ACE2 and increases the expression of ACE1, worsening the imbalance of the RAS. This is quite interesting, since we and others found an increase in Ang I production in patients with severe forms of COVID-19 [83,86]. This evokes the progressive establishment of a process of self-amplification in which the increase in ACE1 makes it possible to accumulate more Ang II, and the absence of hydrolysis of this compound by ACE2 makes it possible to maintain high levels of Ang II in the plasma of COVID-19 patients.

## 6. Pathological Consequences of Ang II-AT1R-HIF-1α Axis Activation in COVID-19

The interaction of SARS-CoV-2 with ACE2 causes ACE2 dysfunction, which results in reduced hydrolysis of Ang II and the overproduction/accumulation of Ang II. Ang II produces its harmful effects through the Ang II-AT1R-HIF-1α axis. HIF-1α was found to be activated in alveolar type 2 cells (the main target for SARS-CoV-2 at the early stage of infection) during acute lung injuries [119]. With the activation of HIF-1α, the transcription of ACE1 mRNA is upregulated in pulmonary artery smooth muscle cells [116]. Moreover, Ang II-induced renal injury requires the activation of HIF-1α [120]. The ACE2 mRNA expression is downregulated after 48 h of hypoxia [121] and the plasma Ang II levels increase under hypoxic conditions [122], worsening the patient’s clinical condition. Moreover, in COVID-19 patients who develop local hypoxia, HIF-1α nuclear translocation stimulates VEGF production, leading to vascular leakage in endothelial capillary cells [123]. Thus, stabilizing HIF-1α could be one of the ways to act on the outcome of COVID-19 patients, reducing hypoxia and acting on ACE2 expression [124].

The accumulation of such physiological disorders may severely impact the prognosis of COVID-19 patients and increase the risk of death. Therapeutic solutions for reducing COVID-19 severity could be found in the pharmacopoeia used by cardiologists to restore the RAS pathway homeostasis. In healthy humans exposed to isocapnic intermittent hypoxia, a significant increase in SaO_2_ was observed, which could be eliminated by treatment with Losartan (a well-known angiotensin II receptor blocker, ARBs, indicated as the first-line antihypertensive drug in patients developing high blood pressure), suggesting a role for AT1R [125]. In addition, AT1R blocking decreases the VEGF expression induced by Ang II [126]. All drugs that have been approved by the Food and Drug Administration (FDA) for treating people with high blood pressure, including renin inhibitors, angiotensin-converting enzyme inhibitors (ACEi), and angiotensin receptor blockers (ARBs), are primarily designed to block or reduce the detrimental effects of Ang II [48,127,128]. Reducing the formation of Ang II by ACEi or blocking the Ang II-AT1R interaction through ARBs may be a suitable strategy for reducing the symptoms of COVID-19 patients [129]. Despite their ability to induce ACE2 expression and potentially promote SARS-CoV-2 infection [130,131], ARBs (e.g., losartan) were found to confer protection against acute lung injury through the reduction in Ang II/AT1R stimulation [132,133]. Previous studies support the beneficial effects of RAS inhibitors in patients with COVID-19 [134,135]. In patients infected with SARS-CoV-2, ARBs are preferred over ACEi for first-line hypertension treatment, and discontinuing treatment is not required [136,137]. Alternatively, the intravenous infusion of 0.4 mg/kg of human recombinant soluble ACE2 (hrsACE2) twice daily may have a beneficial effect [86].

## 7. The Involvement of Cells, Tissues, and Organs in ‘Silent Hypoxia’

One can intuitively conceive that there is huge spatiotemporal heterogeneity in organ involvement in silent hypoxia, presumably due to the fact that certain organs are exposed in priority to SARS-CoV-2 infection and because multiple pathophysiological mechanisms may be causally involved in the early process of local hypoxia. The main cellular receptor to SARS-CoV-2, ACE2, is expressed in virtually all organs, with higher levels in organs that are richly vascularized, such as the lungs, heart, and kidneys [70,138,139]. In the upper airway, ACE2 expression is highest within regions of the sinonasal cavity and pulmonary alveoli and in the lung parenchyma [5,140]. Remarkably, in the respiratory tract, ACE2 is found on a small subset of alveolar type II epithelial cells thought to be a main target for SARS-CoV-2 [5,6]. These cells share the same basement membrane with closely apposed capillary endothelial cells, also expressing high ACE2 levels. It indicates that alveolar type II epithelial lung cells together with the related capillary endothelium may be a primary site of SARS-CoV-2 infection [141]. Consequently, these cells can be destroyed during viral replication and local tissue damage. In response to SARS-CoV-2 infection of pneumocytes, a process of inflammation is activated, which leads the production of interferon associated with the infiltration of monocyte/macrophages and neutrophils in the lung parenchyma [142]. In addition, the increase in Ang II may have variable effects depending on the specialization of the cells being activated via AT1R (e.g., pneumocytes and immune response cells). Ang II has a significant effect on platelets and the coagulation/fibrinolytic system and causes a mild activation of the coagulation cascade through increased plasma levels of the thrombin–antithrombin complex and prothrombin [143,144,145,146]. Moreover, ACE2 catalyzes the conversion of Ang I to Ang-(1-9), the function of which is to reduce plasminogen activator and control fibrinolysis [147]. Finally, SARS-CoV-2 can directly activate platelets by binding to platelet ACE2 [148]. Although patients have relatively well-preserved lung compliance, they experience a compromised pulmonary gas exchange, which leads to a reduced level of oxygenation in the body’s tissues.

The initial clinical presentation of SARS-CoV-2 infection consists of respiratory symptoms such as dry cough, rhinitis, shortness of breath, and, additionally, fever, myalgia, and fatigue. The phase of ‘silent hypoxia’ is a transitional phase during which the more serious pathological consequences of infection are not directly accessible during the clinical examination of the patient. The early pathological manifestation of COVID-19 has a strong vascular component, with exacerbates effects on the microvasculature comprising the arterioles, capillaries, venules, and microthrombosis events. Upon admission of patients, elevated D-dimers (which is a biomarker of the degradation of fibrin and hypercoagulation) highlights a situation of hypercoagulation and pulmonary embolism usually associated with increased mortality in severe COVID-19 patients [32,33]. Increased circulating levels of Ang II are associated with pulmonary inflammation, arterial hypertension, and accelerates thrombosis in arterioles by activating the coagulation cascade and the platelet-derived growth factor (PDGF) [50]. This pattern is detected by peripheral chemical and mechanical sensory receptors located in the aortic arch and the carotid arteries and pulmonary alveolar walls involved in the control of breathing. The gradual increase in the frequency of microvascular thrombi over time leads to a sudden ‘hypoxic spillover’, which initiates the hypoxia phase in COVID-19 patients, since the thrombi prevent gas exchange in the oxygenated areas of tissues. Information from chemical and mechanical sensors together with information from mechanoreceptors located on ribs and muscle tendons are integrated to provide a sensation of dyspnea [149]. Once the system spins out of control, leading to severe COVID-19 and sometimes patient death, the analysis of autopsy lung specimens from COVID-19 patients reveals evidence of inflammatory perivascular lymphocyte infiltration; it documents the existence of microvascular thrombi containing platelets, fibrin, and numerous neutrophil extracellular traps (NETs) releasing [150,151,152]. Deposits of complement components C3, C4d, and C5b-9 were found in the microvasculature of the lungs [12]. Microangiopathic vessel occlusions and endothelium damage has been observed in the kidneys [153]. In severe COVID-19, heart disease (e.g., cardiomyopathy or myocardial infarction) as well as other organ pathologies can be observed but remain late with respect to the onset of ‘silent hypoxia’.

## 8. Discussion

It is likely that the so-called ‘silent hypoxia’ state corresponds to a situation that does not immediately translate clinically (e.g., shortness of breath and dyspnea), but which is expressed by the occurrence of biological disorders, including: (i) an imbalance of the RAS; (ii) an overproduction of Ang II; and (iii) decreased oxygen saturation readings by pulse oximetry.

**Figure 4:**
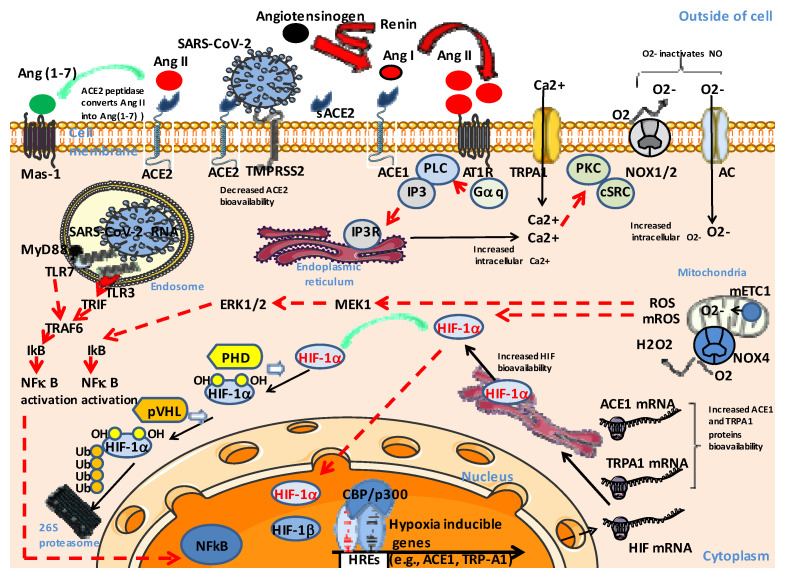
Proposed model of signaling in SARS-CoV-2-induced silent hypoxia. In order to simplify the representation of the signaling pathways, the effects of Ang II have been summarized on a single cell, but several different cell types are involved in the process of ‘silent hypoxia’ and can therefore respond in a specific way to Ang II stimulation. When SARS-CoV-2 is present, the binding of SARS-CoV-2 to ACE2 leads to the dysfunction of ACE2, reduced hydrolysis of Ang II, and increased levels of Ang II. Under this condition, Ang-(1-7) is not sufficient to counterbalance the activity of Ang II. The binding of Ang II to the AT1R activates a signaling cascade that contributes to the lowering of intracellular oxygen and a reduction in the hydroxylation of HIF-1α by PHD. Under these conditions, HIF-1α translocates to the cell nucleus, forms heterodimers with the HIF-1α, and controls the expression of hypoxia-inducible genes. This leads to the upregulation of the ACE1 gene, which contributes to further increases in Ang II, the increased expression of TRPA1, and to the modulation of expression of various genes. In addition, chronic HIF triggers the downregulation of the ACE2 gene and the activation of ADAM17, which leads to the cleavage of the ACE2, the release of soluble ACE2 (sACE2), and S1/sACE2 complexes formation. Finally, there is a decrease in nitric oxide (NO) bioavailability, since O_2_- inactivates NO.

Regarding cells, we postulate that ‘silent hypoxia’ corresponds to an activation of the Ang II-AT1R-HIF-1α axis (Figure 4). When SARS-CoV-2 infects a person, the binding of SARS-CoV-2 to ACE2 leads to the dysfunction of ACE2, reduced hydrolysis of Ang II, and increased levels of Ang II. Under this condition, Ang-(1-7) is not sufficient to counterbalance the activity of Ang II. At the single cell level, the binding of Ang II to the AT1R activates phospholipase C (PLC) via the stimulation of G proteins, resulting in the activation of protein kinase C (PKC) in a calcium-dependent manner (and c-Src), which in turn activates NADPH oxidases. NADPH oxidase (NOX) is involved in superoxide (O_2_-) production. An increase in cytoplasmic Ca^2+^ through the anion channel (AC) favors the NOX4-induced generation of hydrogen peroxide (H_2_O_2_), another reactive oxygen species (ROS). It also stimulates the production of ROS by mitochondria and modulates the activity of mitochondrial electron transport chain complex I (mETC-1) producing O_2_-. This contributes to the lowering of intracellular oxygen and a reduction in the hydroxylation of HIF-1α by prolyl hydroxylase (PHD), which, under normal conditions, is followed by polyubiquitinylation and proteosomal degradation. It also inhibits the hydroxylation of the TRPA1 transmembrane transporter channel, which leads to increased intracellular Ca^2+^. Under these conditions, HIF-1α translocates to the cell nucleus, forms heterodimers with the HIF-1α, and controls the expression of hypoxia-inducible genes. This leads to the upregulation of the ACE1 gene, which contributes to further increases in Ang II, the increased expression of TRPA1, and to the modulation of expression of various genes. In addition, chronic HIF triggers the downregulation of the ACE2 gene and activation of the metalloproteinase ADAM17, which leads to the cleavage of the ACE2 protein with the release soluble ACE2 (sACE2) and the formation of S1/sACE2 complexes [42,65]. Finally, there is a decrease in nitric oxide (NO) bioavailability, since 02- inactivates NO (Figure 4).

Regarding tissues and organs, the activation of the Ang II-AT1R-HIF-1α axis for an increasing number of cells over time leads to the development of local tissue hypoxia. Hypoxia decreases pulmonary NO and causes vasoconstriction [154]. Notably, the inhalation of NO is known to improve Sa0_2_ in patients with hypoxic pulmonary vasoconstriction [155]. Peripheral chemical and mechanical sensory receptors located in the aortic arch, the carotid arteries, and the pulmonary alveolar walls, together with mechanoreceptors located on the ribs and muscle tendons, provide signals integrated by the central respiratory pattern generator complex to adapt the cardiovascular response and control of breathing.

**Figure 5:**
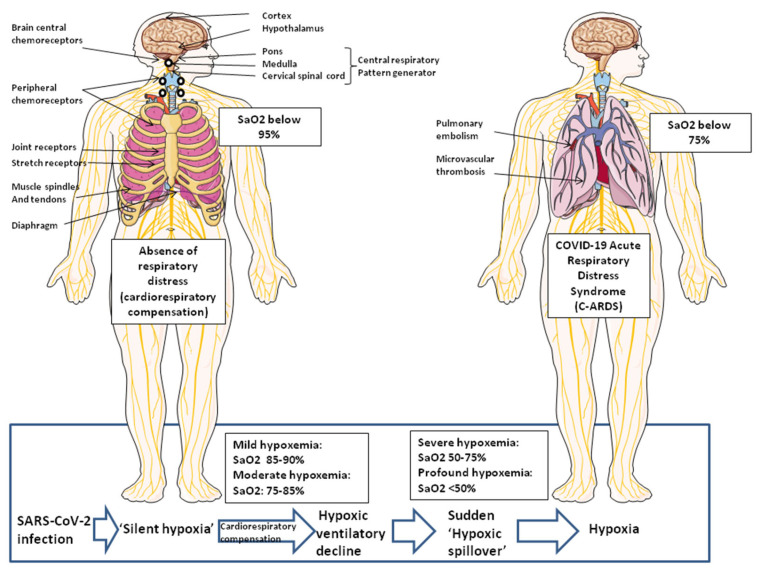
Physiologic mechanisms governing the control of breathing. Peripheral chemical and mechanical sensory receptors are involved in the control of breathing and the sensation of dyspnea. Peripheral chemoreceptors located in the aortic arch and the carotid arteries act as sensors for both O_2_ tension and CO_2_ tension. Pulmonary alveolar walls receptors include joint receptors and stretch receptors. Chemoreceptors in the central and peripheral airways act as irritant sensors. Mechanoreceptors located on the ribs provide information regarding displacement, while muscle tendons provide information regarding tension development. Muscle spindles provide integrated information. The central nervous system integrates these signals and governs breathing (neurons that regulate breathing are widely dispersed in the central cortex, the hypothalamus, the limbic/paralimbic system, pons, and medulla). In moderate hypoxemia, patients respond with intense cardiovascular response (e.g., increased tachycardia, cardiac output, and systemic arterial blood pressure) and accelerated breathing. In contrast, profound hypoxemia (SaO_2_ below 50%) is associated with cardiovascular collapse that results in loss of consciousness, bradycardia, and shock. The ‘hypoxic spillover’ corresponds to sudden deterioration in both oxygen saturation and cardiovascular compensation. For more details, see reference [149].

Regarding the physiopathology of SARS-CoV-2-infected patients, the virus provokes a molecular disease involving ACE2 downregulation. The most important dysfunctional signaling pathway associated with ‘silent hypoxia’ is related to the RAS dysfunction (ACE1/ACE2 ratio imbalance and abnormal increase in Ang II levels) with its share of adverse consequences on the regulation of blood pressure and coagulation (Figure 5). A computer modeling study [41] revealed that silent hypoxia is likely caused by a combination of biological mechanisms that may occur simultaneously in the lungs of COVID-19 patients. A mismatched air-to-blood flow ratio in parts of the lung that do not appear injured on lung scans (as observed in many respiratory illnesses) starts the process of silent hypoxia aggravated by the presence of blood microclots too small to be seen on medical scans, which can form inside the lungs during the step of inflammation and early tissue damage. The blood flow would indeed have to be much higher than normal in areas of the lungs that can no longer gather oxygen, contributing to lower levels of oxygen throughout the entire body. While patients may initially achieve cardiorespiratory compensation to hypoxemia, this compensation can fail precipitously. Herman and colleagues [41] suggested that the lungs of these COVID-19 patients had lost the ability of restricting blood flow to already damaged tissue, and in contrast, were potentially opening up those blood vessels even more, something that is hard to see or measure on a CT scan. Moreover, it has been reported that a shunt mechanism by intrapulmonary vascular dilatation (IPVD) is present among patients with severe COVID-19 [156]. We assume that in some patients, a sudden ‘hypoxic spillover’ marking the tipping point between ‘silent hypoxia’ and hypoxia leads to localized tissue hypoxia, which may have a tendency to become generalized and leads to a sensation of dyspnea. At this point, tissue damage at the level of one or more organs may gradually reveal itself clinically as COVID-19-related acute respiratory distress syndrome (C-ARDS). In the absence of rapid clinical care using forced ventilation with nitric oxide gas and treatment with anti-coagulants to prevent microthrombi, multifocal tissue damage in the pulmonary alveoli and their microcirculatory environment, the primary cellular insults linked to the extent of the subsequent microvascular thrombo-inflammatory response pose major risks, and these patients may quickly progress to severe COVID-19 (Figure 5).

The characterization of the central role of the Ang II-AT1R-ROS-HIF axis in cells that predisposes to the harmful effects of ACE1/ACE2 imbalance and Ang II accumulation, as well as the pharmacological modulation of this signaling pathway, will likely contribute to preventing SARS-CoV-2-positive patients from entering a spiral of deteriorating clinical state. So far, we cannot conclude whether or not having a ‘silent phase’ matters for the Ang II-AT1R-ROS-HIF axis following SARS-CoV-2 infection, but a clinical comparison of survival/mortality in patients with silent hypoxia (phenotype I) and patients with symptomatic hypoxia and clinical signs of acute respiratory failure (phenotype II) indicated higher ICU mortality (29.2% vs. 18.8%, *p* < 0.014) and in-hospital mortality (32.5% vs. 18.8%, *p* < 0.002) in phenotype II (however, a multivariate analysis showed that the cause of silent hypoxia was not clearly identified) [157].

In patients with hypertension hospitalized for COVID-19, a systematic review and meta-analysis suggest that the prior use of RAS inhibitors is associated with a lower risk of mortality, and this has led to the recommendation (current treatment guidelines) that patients taking RAS inhibitors to manage hypertension should continue their treatment when infected by SARS-CoV-2 [46,158]. In addition to the now classic approach of patient ventilation as soon as oxygen saturation monitored by pulse oximetry indicates that SpO2 is 94% or below on two occasions, identifying the cause of ‘silent hypoxia’ at the molecular level makes it possible to consider other early therapeutic strategies that can benefit patients, such as ACEi, ARBs, human recombinant soluble ACE2 (hrsACE2) and, possibly in the future, molecules acting on HIF-1α homeostasis. Notably, Wing and colleagues reported that hypoxia and the HIF prolyl hydroxylase inhibitor FG4592/Roxadustat reduce ACE2 expression and inhibit SARS-CoV-2 entry and replication in lung epithelial cells via an HIF-1α-dependent pathway [159]. They also reported that the pharmacological activation of HIF with FG-4592/Roxadustat significantly reduced epithelial damage and respiratory symptoms in a Syrian hamster model of SARS-CoV-2 infection and increased the expression of ciliated cells [160]. However, care must be taken with this type of molecule because of the pleiotropic role of activated HIF-1α. Indeed, adverse effects of HIF-1α associated with activation on infection and pro-inflammatory responses, epileptogenesis, and pronounced and persistent defects of B cells in COVID-19 patients have also been reported [161,162,163]. This discrepancy between the beneficial and harmful effects of HIF-1α activation in COVID-19 may be due to cell-specific HIF signaling during SARS-CoV2 infection.

COVID-19 is driving large numbers of severely hypoxemic patients to medical care and highlighting the phenomenon of ‘silent hypoxia’ during the early phase of the disease. Here, we attempted to specify how molecular disorders at the level of the cell, followed by tissue damage, lead to a sudden transition from compensated to poorly compensated cardiovascular adaptation to hypoxemia at the level of the infected individual, a phenomenon we named ‘hypoxic spillover’. The analysis of the molecular process that leads to C-ARDS makes it possible to imagine new therapeutic approaches in the treatment of COVID-19 which could be added to the panoply of therapeutic strategies currently available to clinicians.

## Data Availability

No new data were created.
